# The role of fat distribution and inflammation in the origin of endometrial cancer, study protocol of the ENDOCRINE study

**DOI:** 10.1371/journal.pone.0276516

**Published:** 2022-10-27

**Authors:** A. A. S. van den Bosch, J. M. A. Pijnenborg, A. Romano, I. S. Haldorsen, H. M. J. Werner

**Affiliations:** 1 Department of Obstetrics and Gynecology, GROW-School for Oncology and Reproduction, Maastricht University Medical Centre, Maastricht, The Netherlands; 2 Department of Obstetrics & Gynaecology, Radboudumc, Nijmegen, The Netherlands; 3 Mohn Medical Imaging and Visualization Center, Haukeland University Hospital/University of Bergen, Bergen, Norway; Monash University / The University of Melbourne, AUSTRALIA

## Abstract

**Background:**

Obesity is a growing problem worldwide, especially in countries with improved socioeconomic circumstances. Also, in the Netherlands the incidence of overweight and obesity is rising. There is increasing evidence on the association between obesity and tumorigenesis. Of all cancer types, endometrial cancer (EC) has the strongest positive correlation with obesity. Obesity is generally defined as a body mass index (BMI) >30, yet does not cover the differences in fat distribution in visceral and subcutaneous compartments. Visceral fat is assumed to be relatively more metabolically active and likely negative prognostic biomarker in non-endometrioid EC. Whereas subcutaneous fat is mainly responsible for oestrogen production through increased aromatase activity.

**Objective:**

The aim of this study is to compare hormone levels and inflammatory markers after bilateral salpingo-oophorectomy (BSO) in obese and non-obese patients. Secondary objectives are to compare the effect of fat distribution and diagnosis (benign vs. EC) on the observed changes in hormone levels and inflammatory markers, and to compare the effect of BSO on menopausal complaints.

**Methods:**

Prospective multicentre observational cohort study. A total of 160 patients will be included, of which 80 patients with a normal BMI (18–25 kg/m2) and 80 patients with an obese BMI >32–35 kg/m2. Preoperative abdominal CT will be performed and fasting venous blood samples are obtained for hormone levels and inflammation markers analysis. During surgery, adipose tissue biopsies of subcutaneous and visceral (omental and intestinal epiploic fat) compartments will be collected and stored fresh frozen. In addition a fasting blood draw six weeks after surgery will be obtained. All subjects will fill in two questionnaires before surgery and one after surgery.

**Discussion:**

We hypothesize that BMI, the type of fat distribution, and possibly the underlying pathology significantly influence in hormone levels, and systemic inflammation changes after BSO. Previous studies have found several clues for a relationship between obesity and endometrial cancer. We expect that our study will contribute to pinpoint the exact differences between ‘healthy obesity’ and ‘unhealthy obesity’ and will help to identify patients that are more at risk of developing cancer (or possibly suffer from other related problems such as cardiovascular problems e.g.).

## Introduction

**Fig 1:**
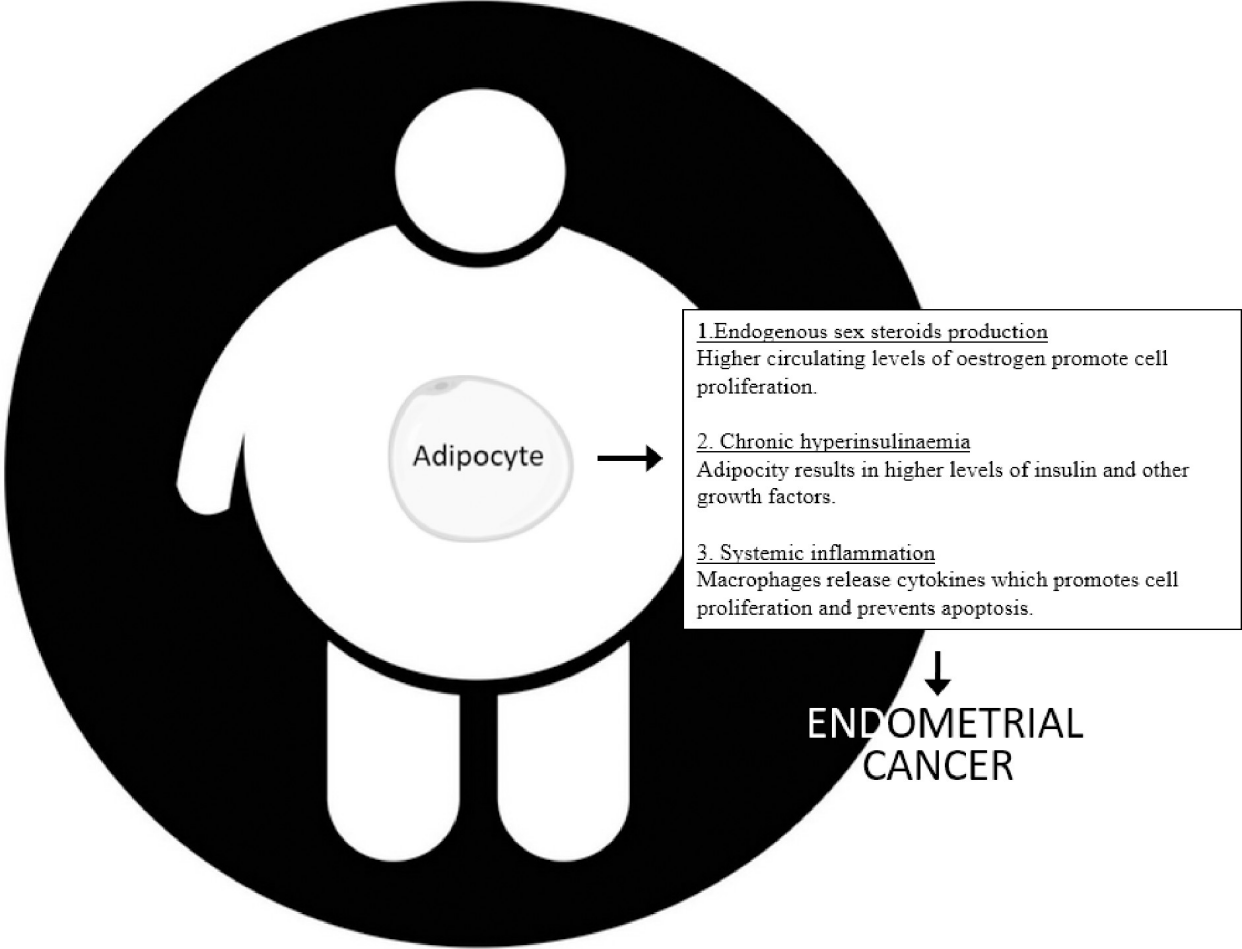
Three main hypotheses for the development of endometrial cancer in obese women.

Obesity is a growing problem worldwide, especially in countries where socioeconomic circumstances improve [1]. In the Netherlands the incidence of overweight and obesity is rising [2]. From the 1990s the percentage of people with a body mass index (BMI) of 25–30 kg/m2 has risen from 24.4% up to 32.4%, and people with a BMI > 30 has more than doubled from 5.6% to 12.1% [2]. Obesity is related to many diseases, including diabetes and cardiovascular disease [3, 4], there is also increasing evidence on the association between obesity and tumorigenesis. The underlying mechanisms linking obesity to cancer development are complex and may not be identical in all tumour types [5, 6]. There are three main hypotheses linking obesity to the development of cancer. 1; Endogenous sex-steroids production, 2; chronic hyperinsulinemia, and 3; systemic inflammation [5, 6] Fig 1. Considering all cancer types, endometrial cancer (EC) incidence has the strongest positive correlation with BMI [7–9]. With every 5 BMI units the risk of EC increases 50% [9]. In EC, long-term unopposed oestrogen stimulation has been established to play a causative role, especially in the aetiology of the endometroid subtype.

Adipose tissue is a very complex endocrine organ that produces adiponectin, leptin, and also steroid hormones. In premenopausal women the ovaries are the main source of oestrogen production [10–12]. However, after menopause, the subcutaneous adipose tissue is the most important producer of oestrogens due to the presence of aromatase, which converts androgens to oestrogens [13]. This mechanism of the production of endogenous sex-steroids plays an important role in the development of EC. Furthermore, obesity-mediated inflammation and insulin resistance have also shown to be important in this process [5, 6, 14]. Chronic hyperinsulinemia, which is common in obese women, has an effect on cell proliferation directly by high insulin levels and indirectly by higher levels of circulating insulin growth factors (IGFs). The chronic inflammation in obese patients results in a higher level of leptin (anti-apoptotic and pro-angiogenetic) and a lower level of adiponectin which as a result promotes cell proliferation and inhibits cell death. These three mechanisms are thought to play an important role in the development of EC [4, 5].

The chronic hyperinsulinemia and systemic inflammation possibly also explains why also for the non-endometrioid subtype, the incidence rate is positively associated with weight gain [15, 16].

Obesity is defined by the world health organisation as a BMI >30 [17]. BMI is a simple, and clinically easily applicable indicator, yet does not cover the complexity of fat distribution in visceral- and subcutaneous compartments, nor the ratio between muscle tissue and fat tissue [16, 18]. Internationally the importance of fat distribution is gaining more attention [19–21].

Previous study showed that a higher visceral fat percentage was a negative prognostic biomarker in non-endometrioid EC (NEEC) [19] compared to subcutaneous fat which is important for oestrogen production through increased aromatase activity [20, 22, 23]. Fat distribution can be measured by imaging methods like CT and MRI or estimated by measurements of skinfolds, DXA-scan and hip-waist circumference. The latter divides women into two groups, women with an apple figure who presumably have more visceral fat and women with a pear figure who most likely have more subcutaneous fat. The MRI an CT related measurements can distinguish between subcutaneous and visceral adipose tissue [5]. Adipose tissue compartments, determined by CT segmentation scans, have been correlated to systemic hormone levels, tumour inflammation and pathway activation in a number of studies [19–22]. In the light of the differences between adipose tissue in the various compartments, the relative increase in central obesity (visceral obesity) through the menopausal transition is of potential additional importance [24].

Through the rising prevalence of obesity worldwide, it is expected that the EC incidence will continue to rise [1, 15, 25, 26]. In the more affluent countries, EC is the most frequent gynaecological malignancy [27, 28]. The primary treatment of EC consists of hysterectomy with BSO. Removal of ovaries serves three goals: 1, it is part of the staging procedure of EC; 2, to exclude the chance of concurrent ovarian cancer and 3, it leads to reduction of the remaining oestrogen and androgen production and thus possibly reduces risk of recurrence. It is uncertain to what extent the sex steroid hormone (and inflammatory) levels drop after BSO, especially in obese women with endometrial cancer. Removal of the ovaries demonstrates to what extent oestrogen production is or was driven by the ovaries or the adipose tissue compartment. Pinpointing this more specifically will help in further understanding the role of adipose tissue in the development of EC.

## Materials and methods

### Study design

This is a prospective observational cohort study. Patients will be recruited from four hospitals including two academic hospitals (academic hospital Maastricht (AZM) and Radboud university medical centre (Radboudumc)) and two large teaching hospitals (Canisius Wilhelmina Ziekenhuis (CWZ) and VieCuri). Inclusions started from 1st of September 2021 in AZM and Radboudumc and inclusions at CWZ and VieCuri will start in 2022. As of this moment the study is actively recruiting. The ethics committee of aZM/UM, Maastricht, approved the study protocol for all participating hospitals, which was registered under NL76255.068.21. All participants will provide written informed consent.

### Objective/aim

The aim of this study is to compare hormone levels and inflammatory markers after BSO in obese and non-obese patients. Secondary objectives are to compare the effect of fat distribution and diagnosis (benign vs. endometrial cancer) on changes in hormone levels and inflammatory markers after BSO in obese and non-obese patients, and to compare the effect of BSO on menopausal complaints. We hypothesize that obese post-menopausal women will still have relatively high circulating concentration of oestrogen, especially women with a high subcutaneous fat percentage.

### Participants (Fig 2)

**Fig 2:**
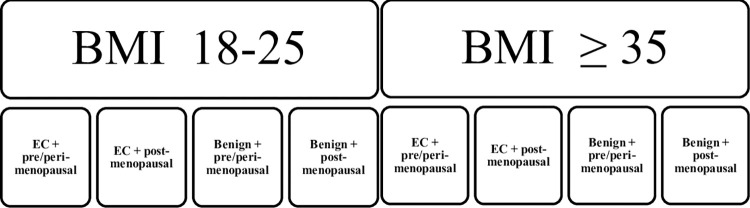
Schematic display of participants. Total number of participants is 160 women.

In total 160 patients will be included, of which 80 patients with a normal BMI between 18–25 kg/m2 and 80 obese patients with BMI ≥35 kg/m2 (WHO obesity class 2). In both these groups an even number of peri-/premenopausal women and postmenopausal women are included, and women with endometrial cancer (EC) and/or atypical endometrial hyperplasia; cases, and women with a benign adnexal pathology (including but not limited to adnexal enlargement/abnormality); controls will be included. Patients will be included pre-operatively. In case of a final diagnosis being ovarian malignancy, this participant will be excluded and a patient with a benign diagnosis included instead.

### Exclusion criteria

Exclusion criteria for this study are another malignancy in the 5 years prior to inclusion (with the exception of basal cell carcinoma), use of systemic hormonal therapy <3 months prior to inclusion, insufficient understanding of the Dutch language, patients not allowed to undergo CT-scan, patients who are expected to be offered HRT immediately after surgery.

### Sample size

There are no previous studies with comparable analysis, allowing for a formal sample size calculation. Therefore, we have taken the difference in hormone level as the starting point for a sample size calculation. Based on an expected difference of 20% in hormone level changes between lean and obese patients (decrease in hormone levels of 70% in lean, and 50% in obese after BSO), using a 90% power and alpha 0.05, 154 patients are required for the primary objective of this study [29]. We have chosen to include 160 women, 80 obese and 80 non-obese. The gap in BMI between the 2 groups (7/10 BMI points) increases the likelihood to obtain significant differences in hormone- and inflammation marker levels.

### Data collection schedule (Fig 3)

**Fig 3:**
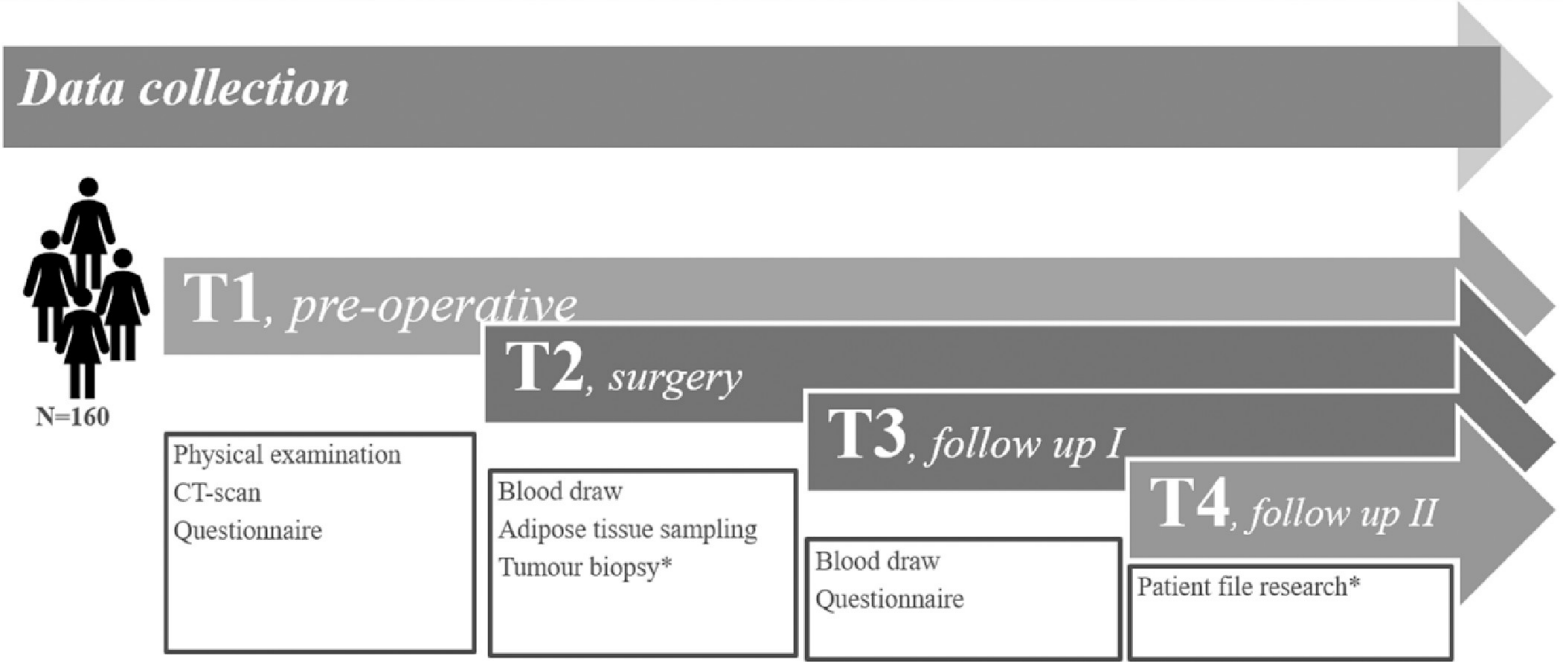
Schematic display of content and timepoints (T) for data collection during the study period. Three time points for patients with benign disease (T1-T3) and four time points (T1-T4) for patients with endometrial cancer (EC).

At three (women with benign diagnoses) or four (women with EC) different time points data will be collected from the subjects.

At timepoint 1 (T1), a physical examination is conducted where hip and waist circumference are measured and patient variables including age, height, weight, BMI, history, cumulative illness rating scale (CIRS) and menopausal status collected. Furthermore, routine abdominal CT imaging will be performed in all patients. Finally, patients are requested to answer a validated questionnaire, the Green climacteric scale enquiring into presence of menopausal symptoms, and are asked to answer three questions on their physical activity level.

At timepoint 2 (T2) subjects will undergo the clinically indicated surgical procedure. At start of surgery, a fasting blood draw will be obtained to measure oestrogen levels and systemic inflammation markers. During surgery subcutaneous adipose tissue and visceral adipose tissue (fat apron and intestinal epiploic fat) as well as tumour (where applicable) will be collected, and snap frozen.

At timepoint 3 (T3) 4 to 6 weeks postoperatively, another fasting blood draw is obtained for measurement of oestrogen levels and systemic inflammation markers. In case of a recent infection the blood draw will be postponed until 10 days after the end of the infection. In case of adjuvant therapy, the postoperative blood draw will be performed latest at start of the adjuvant therapy. Finally, subjects will be asked to fill in the Green climacteric scale questionnaire a second time.

For subjects with the diagnosis of EC clinical follow-up data will be collected during 3 years (T4).

### Data analyses

Using the abdominal CT-scans, abdominal fat volumes will be segmented and quantified using a semi-automatic dedicated software (iNtuition, TeraRecon Inc. San Mateo, CA, USA) [20, 30]. This allows quantification of subcutaneous abdominal fat volume (SAV), visceral abdominal fat volume (VAV), total abdominal fat volume (TAV) and visceral fat percentage (VAV% = [VAV/TAV] x100). Hormone levels (locally in the adipose and tumour tissue and systemically in the blood) will be measured by Liquid-Chromatography-tandem-Mass-Spectrometry (LC-MS/MS) analysis and compared pre- and postoperatively. The biospecimens will be temporarily stored prior to use in this study at the MUMC Biobank [31]. In the Biobank Information System (BIS) type, date and volume of each specimen is recorded. A selection of steroid hormones including oestrogens, androgens, progestogens and corticosteroids as well as local adiponectin, resistin, leptin will be determined in the collected adipose tissue. Inflammatory markers both locally in fat tissue (macrophages, T cells: TNF-α, IL-6, and IL-1β) and systemically (blood) (including C-reactive protein (CRP), interleukin 1 beta (IL1β), interleukin 6 (IL6), tumour necrosis factor alpha (TNFα), insulin-like growth factor 1(IGF1)), will be determined. Samples will be further characterised by omics technologies (genomics, transcriptomics, proteomics and metabolomics). Generated data will be used for biomarker discovery, this data will be used to explore the cellular pathways underlying the various patient characteristics. Furthermore, we will analyse the effect of CT derived fat distribution markers on hormonal/inflammatory activity in different corresponding fat samples.

Pre- and postoperative questionnaires will be analysed and compared. The Green climacteric Scale, assessing menopausal complaints, can be divided into three subcategories; psychological, physical and vasomotor complaints [32]. We will assess all three subcategories separately to evaluate if there are any differences in the groups of patients (pre vs. postmenopausal and obese vs. non-obese patients).

### Stratification

We strive to include a safety margin of 5–10% per stratum to allow for missing data. In both cohorts, due to a short preoperative waiting list (<4 weeks) it is not expected any patients are included whose data or tissue is not subsequently used. For this study, we can optimally compare the included participants using our 20/20/20/20 distribution. Using a population-based inclusion, we would likely have too low numbers in some groups to allow for meaningful analyses. However, as a consequence the external validity is somewhat reduced.

### Statistical analysis

Variables will be analysed quantitatively (CT derived markers, hormones and inflammatory markers) or qualitatively (menopausal symptoms) and stratified by menopausal status and BMI. Comparison of characteristics and study variables will be performed using Chi squared tests (categorical data) and Mann Whitney U test or student t-test (continuous variables, depending on normality of distribution). Correlations will be assessed using Spearman’s rank correlation. Study-related, the oncological patients will be followed for three years for disease recurrence and/or disease-related death. Additionally, correlations between inflammatory markers in adipose tissue and circulating oestrogen levels after treatment and outcome are observed.

## Discussion

The goal of this prospective cohort study is to explore the differences between obese and non-obese women as well as the importance of fat distribution in relation to EC. This study set up, with two academic hospitals and two big teaching hospitals, is well suited to better understand the relation between obesity and endometrial cancer. Because we will include a big range of women with different BMI, menopausal status and pathology, we think this is the perfect setup to generate those much needed knowledge.

A strength of this study is that the big difference in BMI between the two groups will optimally allow for finding any existing obesity-related differences in hormone levels and inflammatory markers. A possible limitation of the resulting BMI gap may thus be the translation of this effect on to the intermediate population. When looking at prevalence of obesity in the Netherlands in 2017, we see that 15.7% of the women were obese, but only 4% had a BMI above 35kg/m2 [33]. However, data from the Netherlands Comprehensive Cancer Organisation specific for women with endometrium carcinoma shows a mean BMI of 30.2 (SD 7.0) kg.m^2^ in 2019 in this population. Of the 2085 EC patients in 2019, 23.4% had a BMI between 30–39.9 and 23.4% a BMI between 25–29.9. It has to be noted that in this dataset BMI was unknown in 35% of the patients [34]. Therefore it is most likely that if we find differences in our population, and further research should confirm this also for the intermediate BMI group (BMI 25–35).

Another strength of this study is that we are able to compare low cost fat distribution measures such as waist-hip circumference with the more accurate fat distribution quantifications based on CT-scan, representing the golden standard for measuring subcutaneous/visceral adipose tissue. Because routine CT imaging, due to cost and radiation is not suitable for application in a healthy general population, waist-hip measurement may represent an easy and attractive alternative to CT if this is correlated to the CT morphometric markers.

Further, by including equally pre/ perimenopausal and postmenopausal women we can pin-point what effect the actual obesity has on the sex-steroid levels. An expected limitations will be that most women will be around menopausal age, because BSO may in specific very low risk situations be refrained from in women <45 years of age, balancing the risks of EC recurrence and iatrogenic menopause and is contra-indicated in women with a benign diagnoses.

Previous studies have found several clues for a relationship between obesity and endometrial cancer [5–7, 14]. We expect that our study will contribute to pinpoint the exact differences between ‘healthy obesity’ and ‘unhealthy obesity’ and will help to identify patients that are more at risk of developing cancer (or possibly suffer from other related problems such as cardiovascular problems e.g.).

Finally, our study will be able to relate the occurrence of menopausal complaints to BMI and fat distribution, which has not been previously addressed in in existing literature.
